# Supplementary material for: The role of REM sleep in neural differentiation of memories in the hippocampus
Source: bioRxiv. 2024 Nov 3:2024.11.01.621588. Preprint. [Version 1] doi: 10.1101/2024.11.01.621588 (PMC11566016; doi:10.1101/2024.11.01.621588)
Supplement: Supplement 1 [file NIHPP2024.11.01.621588v1-supplement-1.pdf]

bioRxiv preprint doi: https://doi.org/10.1101/2024.11.01.621588; this version posted November 3, 2024. The copyright holder for this preprint (which was not certified by peer review) is the author/funder, who has granted bioRxiv a license to display the preprint in perpetuity. It is made available under aCC-BY 4.0 International license.

Supplementary Information

Supplementary Methods

fMRI Session 2: Reward learning task. The reward association task was based on the task used by Wimmer and Shohamy (2012). Below we detail how the A scene from each pair was explicitly associated with either a reward or neutral outcome followed by a test of generalization of the learned reward value to the B scene pairmate. This differs from Wimmer and Shohamy (2012), who explicitly associated the reward/neutral outcomes with their "B" item and tested generalization to their "A" item.

Participants completed two cycles of the task; each cycle included three sub-tasks (one run of the familiarization task, two runs of the reward prediction task, and one run of the decision task). We split our scene pairs from session 1 into two separate sets; each set consisted of 48 scene pairs, with 24 pairs coming from each task condition (violation and nonviolation). The task proceeded in two separate cycles, with one set assigned to each cycle. A full cycle of the task was completed before beginning the second cycle.

During the familiarization task, participants were exposed to associated reward or neutral outcomes for each pair's A scene; no B scenes appeared during this phase. Participants were explicitly instructed to try to learn whether each scene was associated with a reward or neutral outcome. Within each of the two original task conditions (violation and nonviolation), half of the A scenes were paired with a reward (A+), and the other half were paired with a neutral outcome (A-). Each A scene was presented once, followed by its associated reward or neutral outcome (either a picture of a $1 bill or a gray rectangle), in a random order. No responses were required during this phase. Each trial consisted of a scene shown for 1000 ms, followed by a blank 500 ms inter-stimulus-interval, and the outcome image for 1000 ms. The inter-trial-interval was 2000 ms. Each run consisted of 48 trials and lasted approximately 4 minutes.

During the reward prediction task, participants saw A scenes and were asked to predict if each scene led to a reward or neutral outcome; B scenes did not appear during this phase. Each A scene was tested two times in each run (four times total) in a pseudo-random order, such that all A scenes were tested once before any scene was tested a second time. After making their prediction, participants received feedback about the actual outcome, and were able to win or lose "bonus points" based on their prediction. Bonus points were assigned following these rules: If a participant predicted reward and the actual outcome was reward, they won +1 point. If a participant predicted reward and the actual outcome was neutral, they lost -1 point. If a participant predicted neutral, they did not win or lose any points, regardless of the actual outcome. This reward structure was aimed at optimizing the learning rate while keeping the false alarm rate low (e.g., net earning would be 0 if participants predicted 'reward' on all trials). Bonus points were converted to bonus monetary compensation at the end of the study. On each trial of the task, a scene image was shown alone for 2500 ms and participants made their prediction response within this timeframe. The scene remained on the screen as the following pieces of feedback were added in sequence: Participants saw a text version of their response (e.g., "You responded REWARD") for 1000 ms, followed by the actual outcome image (a $1 bill or gray rectangle) for 1000 ms, followed by their winnings (either "You win +1" in green text, or "You lose -1" in red text, or "0" in black text if they did not win or lose points) for 1000 ms. The inter-trial-interval was 500 ms. Each run consisted of 96 trials and lasted approximately 10 minutes.

During the decision task, we assessed preference for A and B scenes. Each decision trial either pitted a rewarded A scene against a neutral A scene (A+ vs. A-), or a rewarded-by-association B scene against a neutral-by-association B scene (B+ vs. B-), and participants were tasked with choosing the scene they thought was more likely to lead to winning a reward. Since B scenes were not shown during the familiarization or reward prediction phases, the reward/neutral status of each B scene was only based on whether its A pairmate was rewarded or not rewarded. Performance was operationalized as the proportion of trials where the rewarded scene (A+ or B+) was chosen (we call this the 'decision score'). We expected these scores to be very high for A+ vs. A- trials due to explicit learning of the A reward associations. Critically, if the learned A+ or A- reward associations generalized to their B scene pairmates, then there should be a preference for B+ scenes (i.e., they should choose the B+ at a rate higher than chance). This is the outcome we predict will be strongest for pairs whose neural representations are more overlapping (i.e., less differentiated). When analyzing the data, pairs that showed a greater decision bias for B+ than A+ (which was a rare occurrence, < 1% of pairs) were excluded from the analysis. Trials with no response were imputed to reflect approximately chance level performance across those no response trials within a participant, rather than removing no response trials from the analysis (i.e., if a participant had an even number of no response trials, half of those trials were randomly assigned an accuracy of 1 and the other half were assigned an accuracy of 0 for an average of 0.5 for those trials; if a participant had an odd number of no response trials, the "extra" trial was assigned an accuracy of 0). This procedure of imputing no response trials was included in our preregistration because we wanted all pairmates to have an equal number of trials represented in the analysis (i.e., we did not

bioRxiv preprint doi: https://doi.org/10.1101/2024.11.01.621588; this version posted November 3, 2024. The copyright holder for this preprint (which was not certified by peer review) is the author/funder, who has granted bioRxiv a license to display the preprint in perpetuity. It is made available under aCC-BY 4.0 International license.

want to remove trials), and it has been argued that always scoring trials with a missing response as 'incorrect' does not accurately reflect memory failures, which at worst should produce 50/50 guessing (Potter et al., 2018). This imputation procedure was employed in 26 participants (range of number of trials with no response in these participants: 1-55); for these 26 participants, their overall accuracy after imputing no response trials was 72.98% vs. 72.34% if we had scored all no response trials as 'incorrect'.

Each A and B scene appeared on four separate decision trials, each time pitted against a different scene counterpart (in other words, all stimulus pairings during the decision phase were unique). However, the pair shufflings were consistent for A and B trial types. For example, if A23+ was pitted against A16- on a trial, then B23+ was pitted against B16- on another trial. Further, the stimulus pairings for each decision trial always came from the same original task condition (violation or nonviolation), and were matched for indoor/outdoor subcategory. The screen location (left or right) of the rewarded image was counterbalanced across the four decision trials for each rewarded image. On each trial of the task, the two scene images were shown together for 2500 ms and participants were instructed to respond within this timeframe. The instructions emphasized that a response should be made on each and every trial, and it was better to guess than not respond at all. Upon a response, a blue frame surrounded the chosen scene image for the remainder of the trial. The inter-trial-interval was 500 ms. Participants earned a bonus point for correctly choosing the rewarded scene on each trial, which was converted to a monetary bonus at the end of the experiment, but feedback about accuracy was not given on a trial-by-trial basis. Each run consisted of 192 trials and lasted approximately 10 minutes.

Supplementary Notes

Supplementary Note 1: Additional preregistered analysis of neural pattern similarity. For analyzing neural pattern similarity, the omnibus test of our full design is a 2x3 mixed-model ANOVA with task condition as the within factor (violation, nonviolation) and group as the between factor (Wake, NREM, REM) (see Figure S3). In our preregistration, we wrote that we expected to find 1) a main effect of task condition, with lower pattern similarity in the violation compared to nonviolation condition, and 2) a significant condition x group interaction. In right CA2/3/DG, there was a main effect of task condition in the predicted direction ($F_{1,66} = 4.17$, $p = 0.045$), but the condition x group interaction was not significant ($F_{2,66} = 2.42$, $p = 0.08$). In left and bilateral CA1, there was a main effect of task condition where pattern similarity was *greater* in the violation than nonviolation condition (left CA1: $F_{1,66} = 6.48$, $p = 0.01$; bilateral CA1: $F_{1,66} = 4.86$, $p = 0.03$), but the condition x group interaction was not significant in either region (left CA1: $F_{2,66} = 0.10$, $p = 0.90$; bilateral CA1: $F_{2,66} = 0.44$, $p = 0.65$). There were no other significant ANOVA results, including no main effects of group.

Supplementary Note 2: Reward learning task behavioral results. After an intial exposure trial, each A scene was tested four times across the reward learning phase. On each trial, participants saw an A scene and predicted if that scene was associated with a reward or neutral outcome. After making their prediction, participants received feedback about the actual outcome. Accuracy increased across the four learning repetitions (main effect of repetition, $F_{3,204} = 215.44$, $p < 0.001$) and was excellent by the fourth and final learning repetition (mean = 0.98, sd = 0.02), indicating that the explicit A scene reward associations were well-learned (Figure S8a). There was no interaction between pair type (violation, nonviolation) and learning repetition ($F_{3,204} = 0.48$, $p = 0.70$; Figure S8b), and no main effect of pair type ($F_{1,68} = 0.52$, $p = 0.47$).

During critical decision test trials, we either pitted a rewarded A scene against a neutral A scene (A+ vs. A-), or a rewarded-by-association B scene against a neutral-by-association B scene (B+ vs. B-). Since B scenes were not shown during the preceding explicit learning phase, the reward/neutral status of each B scene was only based on whether its A pairmate was rewarded or not rewarded. Participants were instructed to choose the scene they thought was more likely to lead to winning a reward. A decision score was computed as the proportion of trials where the rewarded scene (A+ or B+) was correctly chosen over the neutral scene. As expected, decision scores were very high for A trials (trials pitting A+ vs. A-; mean = 0.98, sd = 0.03; Figure S8c) and were near chance for B trials (trials pitting B+ vs. B-; mean = 0.50, sd = 0.05; Figure S8d). Mixed-model ANOVAs with task condition (violation, nonviolation) as the within-factor and group (Wake, NREM, REM) as the between-factor confirmed no significant main effects or interactions for either A trial or B trial decision scores (all *p*s > 0.20). We considered a decision score significantly greater than chance (0.50) on B trials as evidence of generalization, but did not observe that in any condition (all *p*s > 0.07).

We anticipated that it might be difficult to observe behavioral effects at the group level, and were interested in relating variance in this behavioral outcome to variance in neural differentiation. We computed the difference in the decision score for violation minus nonviolation pairs in each participant (Figure S8e); negative values indicate less generalization for violation compared to nonviolation pairs, in line with our hypothesis. We then correlated this behavioral measure with the neural differentiation score in right CA2/3/DG across participants. We predicted a

bioRxiv preprint doi: https://doi.org/10.1101/2024.11.01.621588; this version posted November 3, 2024. The copyright holder for this preprint (which was not certified by peer review) is the author/funder, who has granted bioRxiv a license to display the preprint in perpetuity. It is made available under aCC-BY 4.0 International license.

positive relationship, such that more negative neural differentiation scores would be associated with more negative behavioral difference scores (i.e., more violation-related neural differentiation, less violation-related generalization). However, we did not observe any significant relationships between our neural and behavioral measures in all participants together ($r = -0.0012$, $p = 0.99$), or within the REM group specifically ($r = -0.16$, $p = 0.47$). As such, we did not proceed with the mediation analysis proposed in the preregistration.

bioRxiv preprint doi: https://doi.org/10.1101/2024.11.01.621588; this version posted November 3, 2024. The copyright holder for this preprint (which was not certified by peer review) is the author/funder, who has granted bioRxiv a license to display the preprint in perpetuity. It is made available under aCC-BY 4.0 International license.

964 # Supplementary Figures

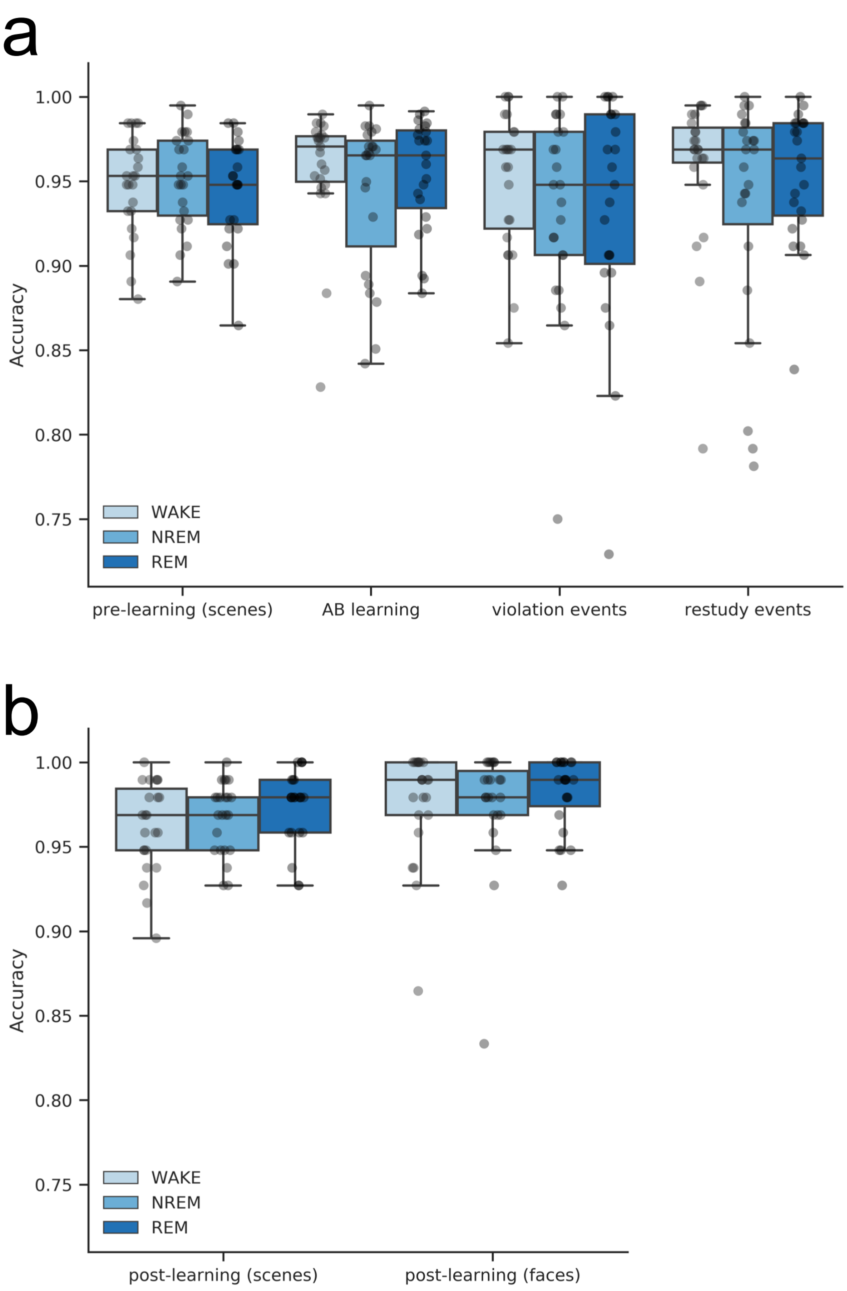

Figure S1. Behavioral performance. (**A**) Accuracy on the session 1 cover task for the following trial types: pre-learning scene snapshots, three repetitions of A-B learning, violation events (seeing faces X or Y instead of scene B), and restudy events (B not following A). (**B**) Accuracy on the session 2 cover task for the post-learning scene and face snapshots. *n* = 23 in each group.

bioRxiv preprint doi: https://doi.org/10.1101/2024.11.01.621588; this version posted November 3, 2024. The copyright holder for this preprint (which was not certified by peer review) is the author/funder, who has granted bioRxiv a license to display the preprint in perpetuity. It is made available under aCC-BY 4.0 International license.

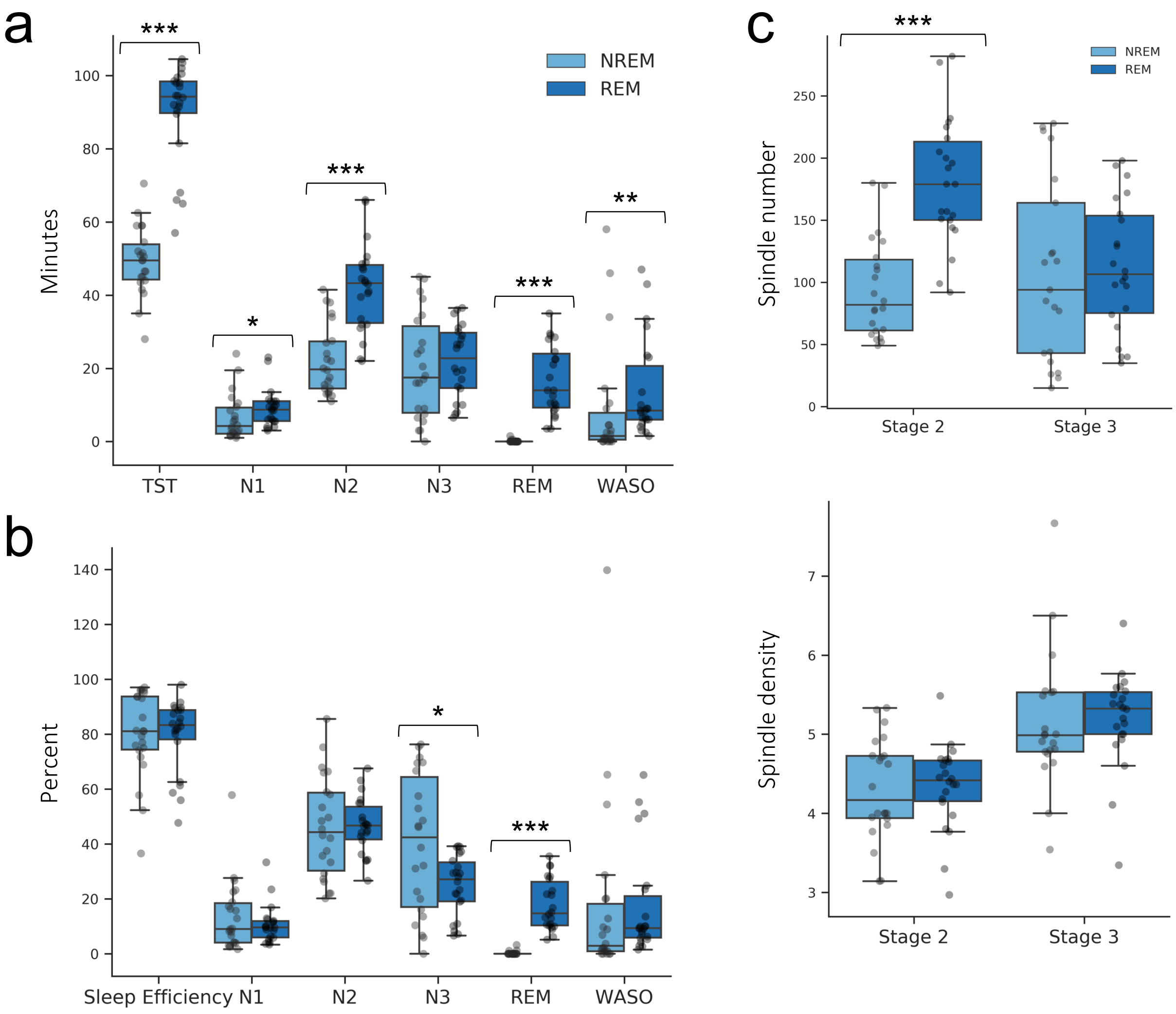

Figure S2. Nap sleep architecture. (**A**) Minutes of total sleep time (TST), Stage 1 (N1), Stage 2 (N2), Stage 3 (N3), REM sleep, and wake after sleep onset (WASO). (**B**) Sleep variables as a percentage of total sleep time. Sleep efficiency is total sleep time/time spent in bed trying to nap. (**C**) Number of spindle events (top panel) and spindle density (bottom panel) in Stage 2 and Stage 3 sleep, derived from channel CPz. $n = 22$ in each group; *$p< 0.05$, **$p< 0.01$, ***$p< 0.001$.

bioRxiv preprint doi: https://doi.org/10.1101/2024.11.01.621588; this version posted November 3, 2024. The copyright holder for this preprint (which was not certified by peer review) is the author/funder, who has granted bioRxiv a license to display the preprint in perpetuity. It is made available under aCC-BY 4.0 International license.

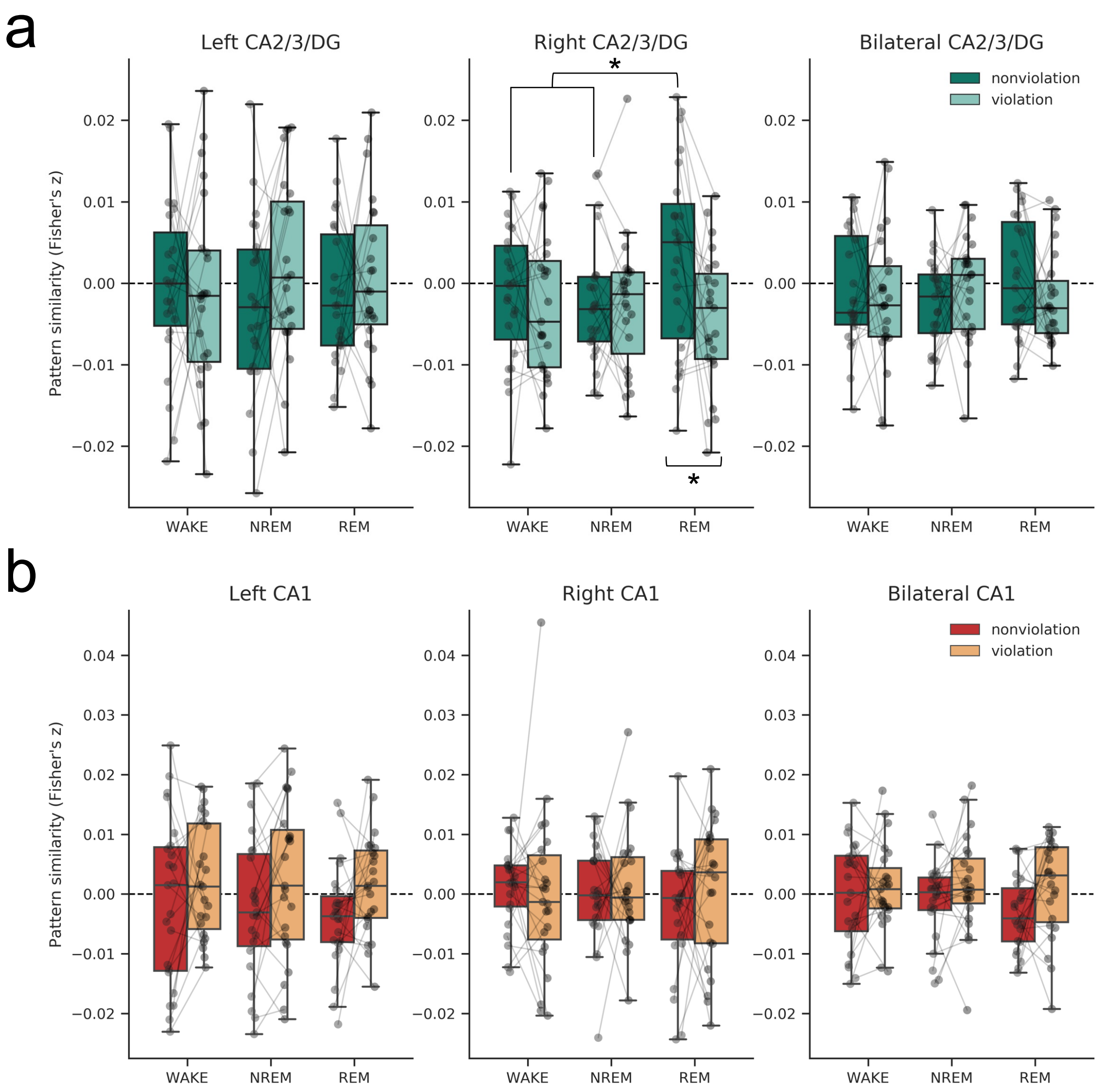

Figure S3. Pattern similarity by task condition in (a) CA2/3/DG and (b) CA1. The REM group showed the predicted pattern of results in right CA2/3/DG, with pattern similarity in the violation condition significantly less than in the nonviolation condition (*p* = 0.02, note this is the same finding depicted in Figure 2a in the main text). An exploratory contrast revealed that nonviolation condition pattern similarity values were significantly higher in the REM group than Wake/NREM groups (*p* = 0.04). *n* = 23 in each group; **p*< 0.05.

bioRxiv preprint doi: https://doi.org/10.1101/2024.11.01.621588; this version posted November 3, 2024. The copyright holder for this preprint (which was not certified by peer review) is the author/funder, who has granted bioRxiv a license to display the preprint in perpetuity. It is made available under aCC-BY 4.0 International license.

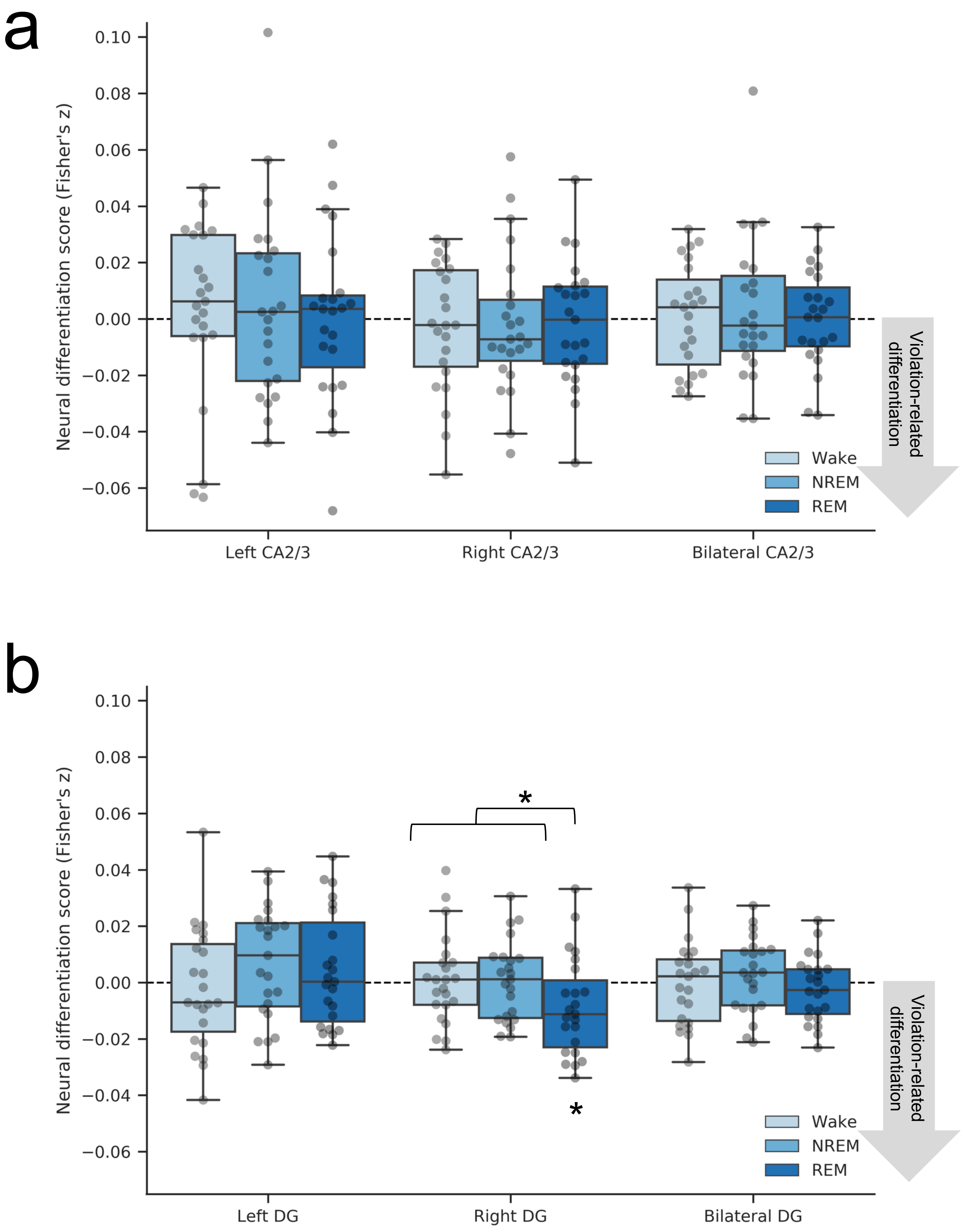

Figure S4. Neural differentiation in (a) CA2/3 and (b) DG separately. Neural differentiation scores are the difference in pattern similarity for the violation minus nonviolation task conditions. A contrast revealed more violation-related neural differentiation in the REM group compared to the Wake and NREM groups in right DG (p = 0.03, not significant following correction for multiple comparisons). Within the REM group, the neural differentiation score was significantly different from zero (p = 0.015, one-tailed) and reliably item-specific based on a randomization analysis (p = 0.02). n = 23 in each group; *p< 0.05.

bioRxiv preprint doi: https://doi.org/10.1101/2024.11.01.621588; this version posted November 3, 2024. The copyright holder for this preprint (which was not certified by peer review) is the author/funder, who has granted bioRxiv a license to display the preprint in perpetuity. It is made available under aCC-BY 4.0 International license.

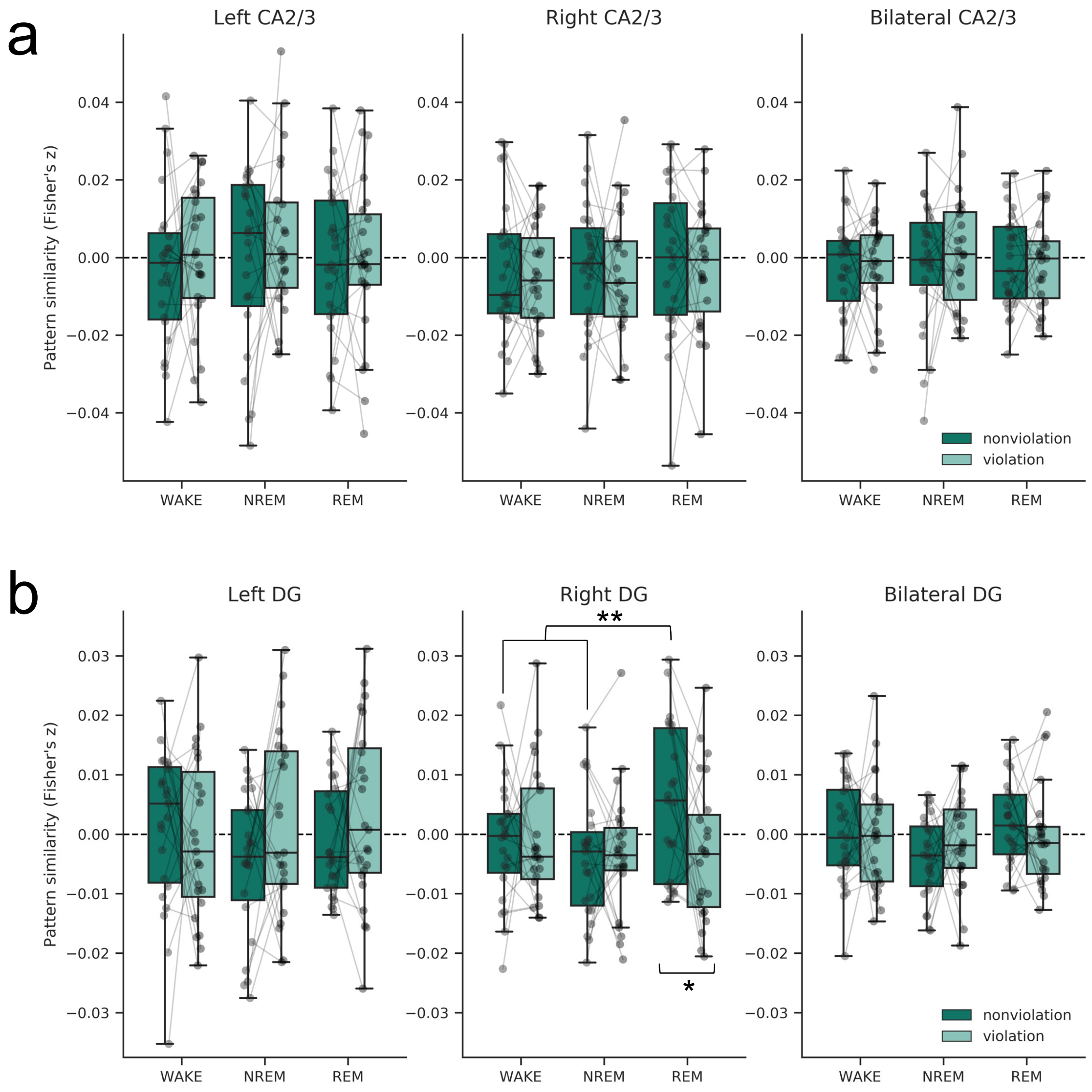

Figure S5. Pattern similarity by task condition in (a) CA2/3 and (b) DG separately. The REM group showed the predicted pattern of results in right DG, with pattern similarity in the violation condition less than in the nonviolation condition ($p = 0.03$, note this is the same finding depicted in Figure S4b). An exploratory contrast revealed that nonviolation condition pattern similarity values were significantly higher in the REM group than Wake/NREM groups ($p = 0.007$). $n = 23$ in each group; *$p < 0.05$, **$p < 0.01$.

bioRxiv preprint doi: https://doi.org/10.1101/2024.11.01.621588; this version posted November 3, 2024. The copyright holder for this preprint (which was not certified by peer review) is the author/funder, who has granted bioRxiv a license to display the preprint in perpetuity. It is made available under aCC-BY 4.0 International license.

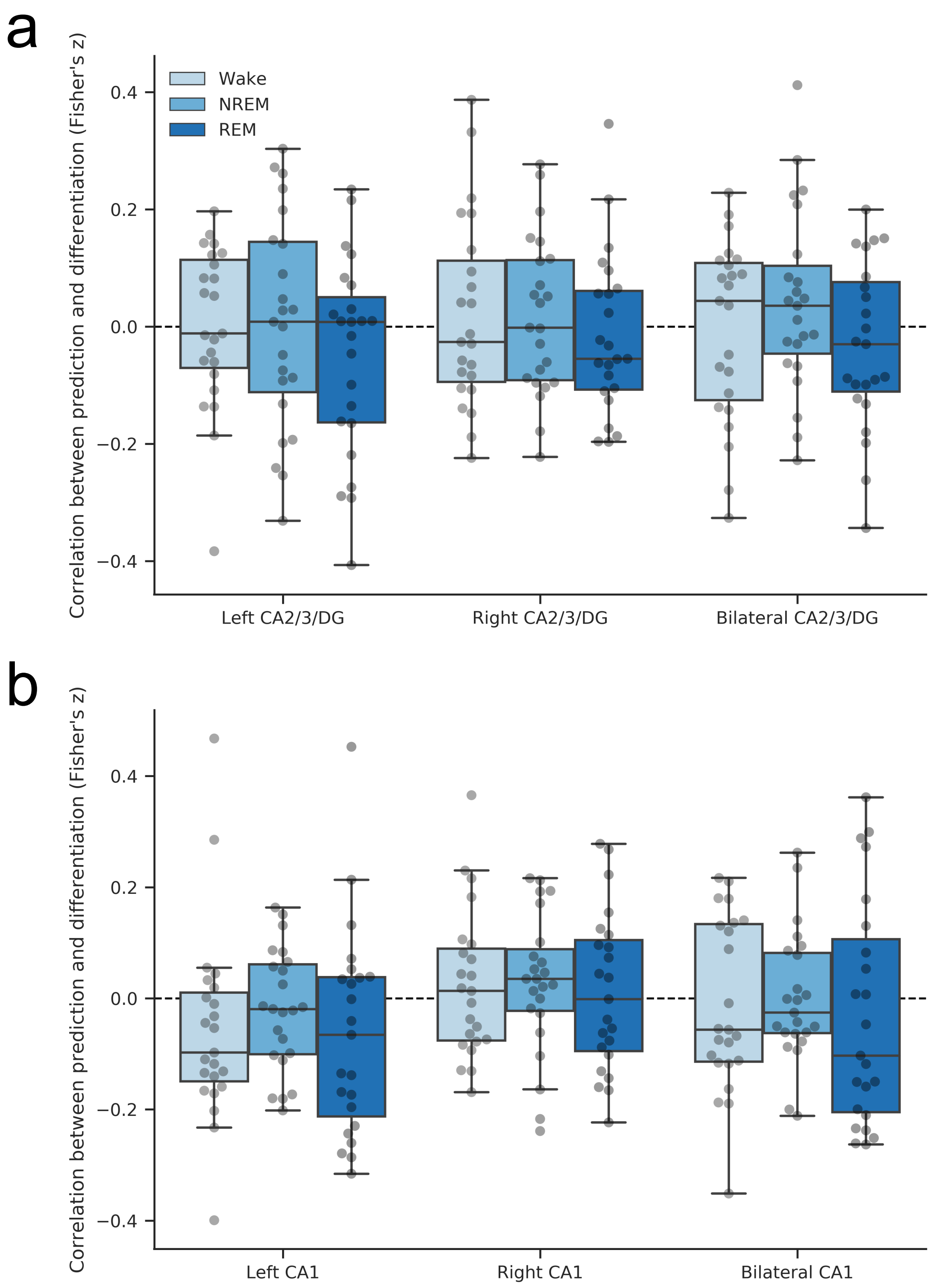

Figure S6. Relationship between prediction and differentiation in (a) CA2/3/DG and (b) CA1. We hypothesized that a negative correlation should exist between prediction and differentiation (i.e., higher levels of B activation during violation events should be associated with decreased A-B pattern similarity). However, there was no reliable relationship between B prediction and neural differentiation in any group in either CA2/3/DG or CA1 ROIs. $n = 23$ in each group.

bioRxiv preprint doi: https://doi.org/10.1101/2024.11.01.621588; this version posted November 3, 2024. The copyright holder for this preprint (which was not certified by peer review) is the author/funder, who has granted bioRxiv a license to display the preprint in perpetuity. It is made available under aCC-BY 4.0 International license.

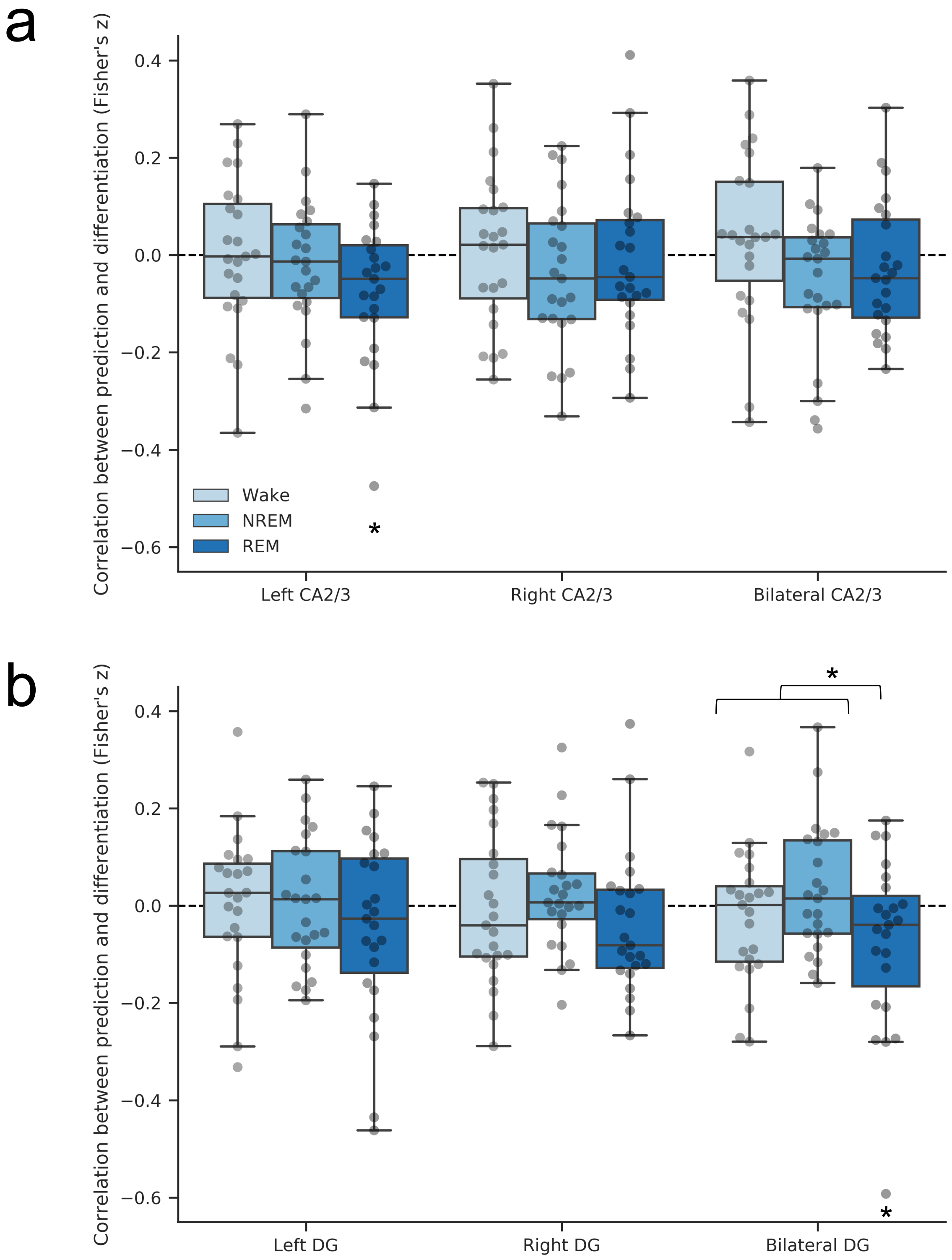

Figure S7. Relationship between prediction and differentiation in (a) CA2/3 and (b) DG separately. A contrast testing for a difference between the REM group and the two other groups showed a difference in bilateral DG (p=0.046, not significant after correcting for multiple comparisons), with the REM group showing the predicted negative relationship between B prediction and A-B pattern similarity (one-sample t-test, p=0.03, one-tailed). The relationship was also significantly different from zero in the REM group in left CA2/3 (p = 0.01, one-tailed). n = 23 in each group; *p< 0.05

bioRxiv preprint doi: https://doi.org/10.1101/2024.11.01.621588; this version posted November 3, 2024. The copyright holder for this preprint (which was not certified by peer review) is the author/funder, who has granted bioRxiv a license to display the preprint in perpetuity. It is made available under aCC-BY 4.0 International license.

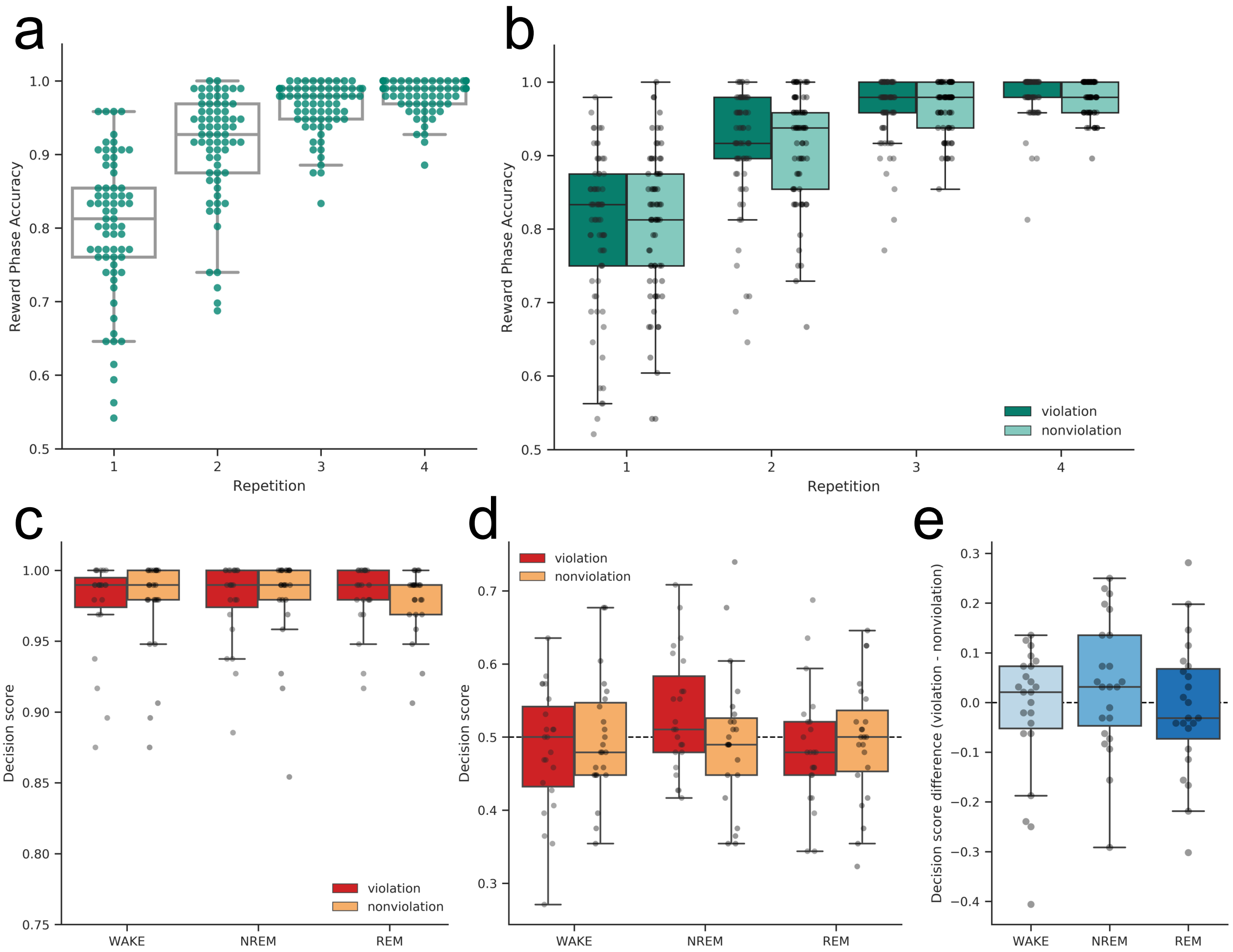

Figure S8. Reward learning task behavioral performance measures. During the reward prediction phase, participants saw an A scene and predicted if that scene was associated with a reward or neutral outcome. (**a**) Reward prediction accuracy increased across four learning repetitions ($p < 0.001$), and (**b**) there was no interaction between pair type (violation, nonviolation) and learning repetition. During the decision phase, participants were shown one rewarded and one neutral scene, and chose the one they thought was most likely to lead to winning a reward. (**c**) The decision score for explicitly-learned A scenes was near 1 for most participants, reflecting the strong learning of A scene reward associations. (**d**) The decision score on B trials was no different than chance in any group or condition, and (**e**) no group showed a difference in decision bias between pair types (violation-nonviolation) significantly different from zero.

bioRxiv preprint doi: https://doi.org/10.1101/2024.11.01.621588; this version posted November 3, 2024. The copyright holder for this preprint (which was not certified by peer review) is the author/funder, who has granted bioRxiv a license to display the preprint in perpetuity. It is made available under aCC-BY 4.0 International license.

References

Potter, K. W., Huszar, L. D., and Huber, D. E. (2018). Does inhibition cause forgetting after selective retrieval? a reanalysis and failure to replicate. *Cortex*, 104:26–45.

Wimmer, G. E. and Shohamy, D. (2012). Preference by association: how memory mechanisms in the hippocampus bias decisions. *Science*, 338(6104):270–273.

bioRxiv preprint doi: https://doi.org/10.1101/2024.11.01.621588; this version posted November 3, 2024. The copyright holder for this preprint (which was not certified by peer review) is the author/funder, who has granted bioRxiv a license to display the preprint in perpetuity. It is made available under aCC-BY 4.0 International license.

Supplementary Table

Table S1. Sleep variables correlated with the neural differentiation score

	CA2/3/DG			CA1		
	left	right	bilateral	left	right	bilateral
TST (min)	–0.087	–0.150	–0.138	–0.028	0.037	0.019
N1 (min)	–0.086	–0.029	–0.092	–0.082	–0.104	–0.140
N2 (min)	–0.055	0.049	0.001	0.104	0.189	0.197
N3 (min)	0.048	–0.170	–0.062	–0.267	–0.145	–0.242
REM (min)	–0.120	0.099	0.011	0.361	–0.018	0.197
N2 spindle density	–0.002	–0.119	–0.084	–0.013	0.120	0.112
N3 spindle density	–0.011	0.170	0.116	0.362*	0.158	0.336*

Note: Values reported are Pearson's r. Spindle densities are from electrode CPz. All correlations include n = 44, except REM minutes is n = 22, and N3 spindle density is n = 43 (one participant had 0 minutes of N3 sleep). *p< 0.05, not corrected for multiple comparisons
